# Supplementary material for: Optimization of infectious bronchitis virus-like particle expression in Nicotiana benthamiana as potential poultry vaccines
Source: PLoS One. 2023 Jul 20;18(7):e0288970. doi: 10.1371/journal.pone.0288970 (PMC10358894; doi:10.1371/journal.pone.0288970)
Supplement: S1 Fig — The murine signal peptide is highlighted in blue, the linker in magenta, and the S2 domain in grey with heptad repeat 1 in yellow, the central helix in green, and the two stabilizing proline substitutions in boldface and underlined. (DOCX) [file pone.0288970.s001.docx]

MGWSWIFLFLLSGAAGVHCNLFDSDNNYVYYYQSAFRPPNGWHLQGGAYAVVNSTNHTSNAGSAQGCTVGVIKDVYNQSVASIAMTAPLQGMAWFCTAYCNFSDTTVFVTHCYHIRISAMKNGSLFYNLTVSVSKYPNFKSFQCVNNFTSVYLNGDLVFTSNKTTDVTSAGVYFKAGGPVNYSIMKEFKVLAYFVNGTAQDVILCDNSPKGLLACQYNTGNFSDGFYPFTNSTLVREKFIVYRESSFNTTLALTNFTFTNVSNAQPNSGGVNTFHLYQTQTAQSGYYNFNLSFLSQFVYKASDFMYGSYHPSCSFRPETINSGLWFNSLSVSLTYGPLQGGCKQSVFSGKATCCYAYSYKGPMACKGVYSGELRTNFECGLLVYVTKSDGSRIQTRTEPLVLTQYNYNNITLDKCVAYNIYGRVGQGFITNVTDSAANFSYLADGGLAILDTSGAIDVFVVQGIYGLNYYKVNPCEDVNQQFVVSGGNIVGILTSRNETGSEQVENQFYVKLTNSSGGGGGSIGQNVTSCPYVSYGRFCIEPDGSLKMIVPEELKQFVAPLLNITESVLIPNSFNLTVTDEYIQTRMDKVQINCLQYVCGNSLECRKLFQQYGPVCDNILSVVNSVSQKEDMELLSFYSSTKPKGYDTPVLSNVSTGEFNISLLLKTPISSSGRSFIEDLLFTSVETVGLPTDAEYKKCTAGPLGTLKDLICAREYNGLLVLPPIITADMQTMYTASLVGAMAFGGITSAAAIPFATQIQARINHLGITQSLLMKNQEKIAASFNKAIGHMQEGFRSTSLALQQIQDVVNKQSAILTETMNSLNKNFGAITSVIQDIYAQLD**PP**QADAQVDRLITGRLSSLSVLASAKQSEYIRVSQQRELATKKINECVKSQSNRYGFCGSGRHVLSIPQNAPNGIVFIHFTYTPESFVNVTAIVGFCVNPANASQYAIVPANGRGVFIQVNGSYYITARDMYMPRDITAGDIVTLTSCQANYVNVNKTVINTFVEDDDFNFNDELSKWWNDTKHELPDFDEFNYTVPVLNISNEIDRIQEVIQGLNDSLIDLETLSILKTYIKWPWYVWLAIFFAIIIFILILGWVFFMTGCCGCCCGCFGIIPLMSKCGKKSSYYTTFDNDVVTEQYRPKKSV
