# Supplementary material for: Optimization of infectious bronchitis virus-like particle expression in Nicotiana benthamiana as potential poultry vaccines
Source: PLoS One. 2023 Jul 20;18(7):e0288970. doi: 10.1371/journal.pone.0288970 (PMC10358894; doi:10.1371/journal.pone.0288970)
Supplement: S2 Fig — Protein confirmation using LC-MS/MS-based peptide sequencing of the modified IBV spike protein constructs compared in this study (A) mIBV-S2P, (B) mIBV-S2P-IAV-H6TM/CT, and (C) mIBV-S2P-NDV-FTM/CT. The percentage sequence coverage is indicated above with several unique peptides identified with ˃ 90% confidence. Peptides with ˃ 95% confidence are highlighted in green, those with 50–95% confidence in yellow, and those with <50% confidence in red. No peptides were identified for the non-highlighted regions of the sequence (grey). (DOCX) [file pone.0288970.s002.docx]

**mIBV-S2P: M: E: N (2:1:1:1)**

**23 % coverage (95 % confidence) with 24 unique peptides**

MGWSWIFLFLLSGAAGVHCNLFDSDNNYVYYYQSAFRPPNGWHLQGGAYAVVNSTNHTSNAGSAQGCTVGVIKDVYNQSVASIAMTAPLQGMAWFCTAYCNFSDTTVFVTHCYHIRISAMKNGSLFYNLTVSVSKYPNFKSFQCVNNFTSVYLNGDLVFTSNKTTDVTSAGVYFKAGGPVNYSIMKEFKVLAYFVNGTAQDVILCDNSPKGLLACQYNTGNFSDGFYPFTNSTLVREKFIVYRESSFNTTLALTNFTFTNVSNAQPNSGGVNTFHLYQTQTAQSGYYNFNLSFLSQFVYKASDFMYGSYHPSCSFRPETINSGLWFNSLSVSLTYGPLQGGCKQSVFSGKATCCYAYSYKGPMACKGVYSGELRTNFECGLLVYVTKSDGSRIQTRTEPLVLTQYNYNNITLDKCVAYNIYGRVGQGFITNVTDSAANFSYLADGGLAILDTSGAIDVFVVQGIYGLNYYKVNPCEDVNQQFVVSGGNIVGILTSRNETGSEQVENQFYVKLTNSSGGGGGSIGQNVTSCPYVSYGRFCIEPDGSLKMIVPEELKQFVAPLLNITESVLIPNSFNLTVTDEYIQTRMDKVQINCLQYVCGNSLECRKLFQQYGPVCDNILSVVNSVSQKEDMELLSFYSSTKPKGYDTPVLSNVSTGEFNISLLLKTPISSSGRSFIEDLLFTSVETVGLPTDAEYKKCTAGPLGTLKDLICAREYNGLLVLPPIITADMQTMYTASLVGAMAFGGITSAAAIPFATQIQARINHLGITQSLLMKNQEKIAASFNKAIGHMQEGFRSTSLALQQIQDVVNKQSAILTETMNSLNKNFGAITSVIQDIYAQLDPPQADAQVDRLITGRLSSLSVLASAKQSEYIRVSQQRELATKKINECVKSQSNRYGFCGSGRHVLSIPQNAPNGIVFIHFTYTPESFVNVTAIVGFCVNPANASQYAIVPANGRGVFIQVNGSYYITARDMYMPRDITAGDIVTLTSCQANYVNVNKTVINTFVEDDDFNFNDELSKWWNDTKHELPDFDEFNYTVPVLNISNEIDRIQEVIQGLNDSLIDLETLSILKTYIKWPWYVWLAIFFAIIIFILILGWVFFMTGCCGCCCGCFGIIPLMSKCGKKSSYYTTFDNDVVTEQYRPKKSV

**mIBV-S2P-IAV-H6^TM/CT^: IAV M2 (2:1)**

**25.8 % coverage (95 % confidence) with 25 unique peptides**

MGWSWIFLFLLSGAAGVHCNLFDSDNNYVYYYQSAFRPPNGWHLQGGAYAVVNSTNHTSNAGSAQGCTVGVIKDVYNQSVASIAMTAPLQGMAWFCTAYCNFSDTTVFVTHCYHIRISAMKNGSLFYNLTVSVSKYPNFKSFQCVNNFTSVYLNGDLVFTSNKTTDVTSAGVYFKAGGPVNYSIMKEFKVLAYFVNGTAQDVILCDNSPKGLLACQYNTGNFSDGFYPFTNSTLVREKFIVYRESSFNTTLALTNFTFTNVSNAQPNSGGVNTFHLYQTQTAQSGYYNFNLSFLSQFVYKASDFMYGSYHPSCSFRPETINSGLWFNSLSVSLTYGPLQGGCKQSVFSGKATCCYAYSYKGPMACKGVYSGELRTNFECGLLVYVTKSDGSRIQTRTEPLVLTQYNYNNITLDKCVAYNIYGRVGQGFITNVTDSAANFSYLADGGLAILDTSGAIDVFVVQGIYGLNYYKVNPCEDVNQQFVVSGGNIVGILTSRNETGSEQVENQFYVKLTNSSGGGGGSIGQNVTSCPYVSYGRFCIEPDGSLKMIVPEELKQFVAPLLNITESVLIPNSFNLTVTDEYIQTRMDKVQINCLQYVCGNSLECRKLFQQYGPVCDNILSVVNSVSQKEDMELLSFYSSTKPKGYDTPVLSNVSTGEFNISLLLKTPISSSGRSFIEDLLFTSVETVGLPTDAEYKKCTAGPLGTLKDLICAREYNGLLVLPPIITADMQTMYTASLVGAMAFGGITSAAAIPFATQIQARINHLGITQSLLMKNQEKIAASFNKAIGHMQEGFRSTSLALQQIQDVVNKQSAILTETMNSLNKNFGAITSVIQDIYAQLDPPQADAQVDRLITGRLSSLSVLASAKQSEYIRVSQQRELATKKINECVKSQSNRYGFCGSGRHVLSIPQNAPNGIVFIHFTYTPESFVNVTAIVGFCVNPANASQYAIVPANGRGVFIQVNGSYYITARDMYMPRDITAGDIVTLTSCQANYVNVNKTVINTFVEDDDFNFNDELSKWWNDTKHELPDFDEFNYTVPVLNISNEIDRIQEVIQGLNDSLIDLETLSILKTYIKLAIYSTVSSSLVLVGLIIAMGLWMCSNGSMQCRVCI

**mIBV-S2P-NDV-F^TM/CT^: NDV matrix (2:1)**

**33.7% coverage at 95% confidence with 37 unique peptides**

MGWSWIFLFLLSGAAGVHCNLFDSDNNYVYYYQSAFRPPNGWHLQGGAYAVVNSTNHTSNAGSAQGCTVGVIKDVYNQSVASIAMTAPLQGMAWFCTAYCNFSDTTVFVTHCYHIRISAMKNGSLFYNLTVSVSKYPNFKSFQCVNNFTSVYLNGDLVFTSNKTTDVTSAGVYFKAGGPVNYSIMKEFKVLAYFVNGTAQDVILCDNSPKGLLACQYNTGNFSDGFYPFTNSTLVREKFIVYRESSFNTTLALTNFTFTNVSNAQPNSGGVNTFHLYQTQTAQSGYYNFNLSFLSQFVYKASDFMYGSYHPSCSFRPETINSGLWFNSLSVSLTYGPLQGGCKQSVFSGKATCCYAYSYKGPMACKGVYSGELRTNFECGLLVYVTKSDGSRIQTRTEPLVLTQYNYNNITLDKCVAYNIYGRVGQGFITNVTDSAANFSYLADGGLAILDTSGAIDVFVVQGIYGLNYYKVNPCEDVNQQFVVSGGNIVGILTSRNETGSEQVENQFYVKLTNSSGGGGGSIGQNVTSCPYVSYGRFCIEPDGSLKMIVPEELKQFVAPLLNITESVLIPNSFNLTVTDEYIQTRMDKVQINCLQYVCGNSLECRKLFQQYGPVCDNILSVVNSVSQKEDMELLSFYSSTKPKGYDTPVLSNVSTGEFNISLLLKTPISSSGRSFIEDLLFTSVETVGLPTDAEYKKCTAGPLGTLKDLICAREYNGLLVLPPIITADMQTMYTASLVGAMAFGGITSAAAIPFATQIQARINHLGITQSLLMKNQEKIAASFNKAIGHMQEGFRSTSLALQQIQDVVNKQSAILTETMNSLNKNFGAITSVIQDIYAQLDPPQADAQVDRLITGRLSSLSVLASAKQSEYIRVSQQRELATKKINECVKSQSNRYGFCGSGRHVLSIPQNAPNGIVFIHFTYTPESFVNVTAIVGFCVNPANASQYAIVPANGRGVFIQVNGSYYITARDMYMPRDITAGDIVTLTSCQANYVNVNKTVINTFVEDDDFNFNDELSKWWNDTKHELPDFDEFNYTVPVLNISNEIDRIQEVIQGLNDSLIDLETLSILKTYIKLITYIVLTIISLVFGILSLILACYLMYKQKAQQKTLLWLGNNTLDQMRATTKM
