# Supplementary figures and images for: Optimization of infectious bronchitis virus-like particle expression in Nicotiana benthamiana as potential poultry vaccines
Source: PLoS One. 2023 Jul 20;18(7):e0288970. doi: 10.1371/journal.pone.0288970 (PMC10358894; doi:10.1371/journal.pone.0288970)

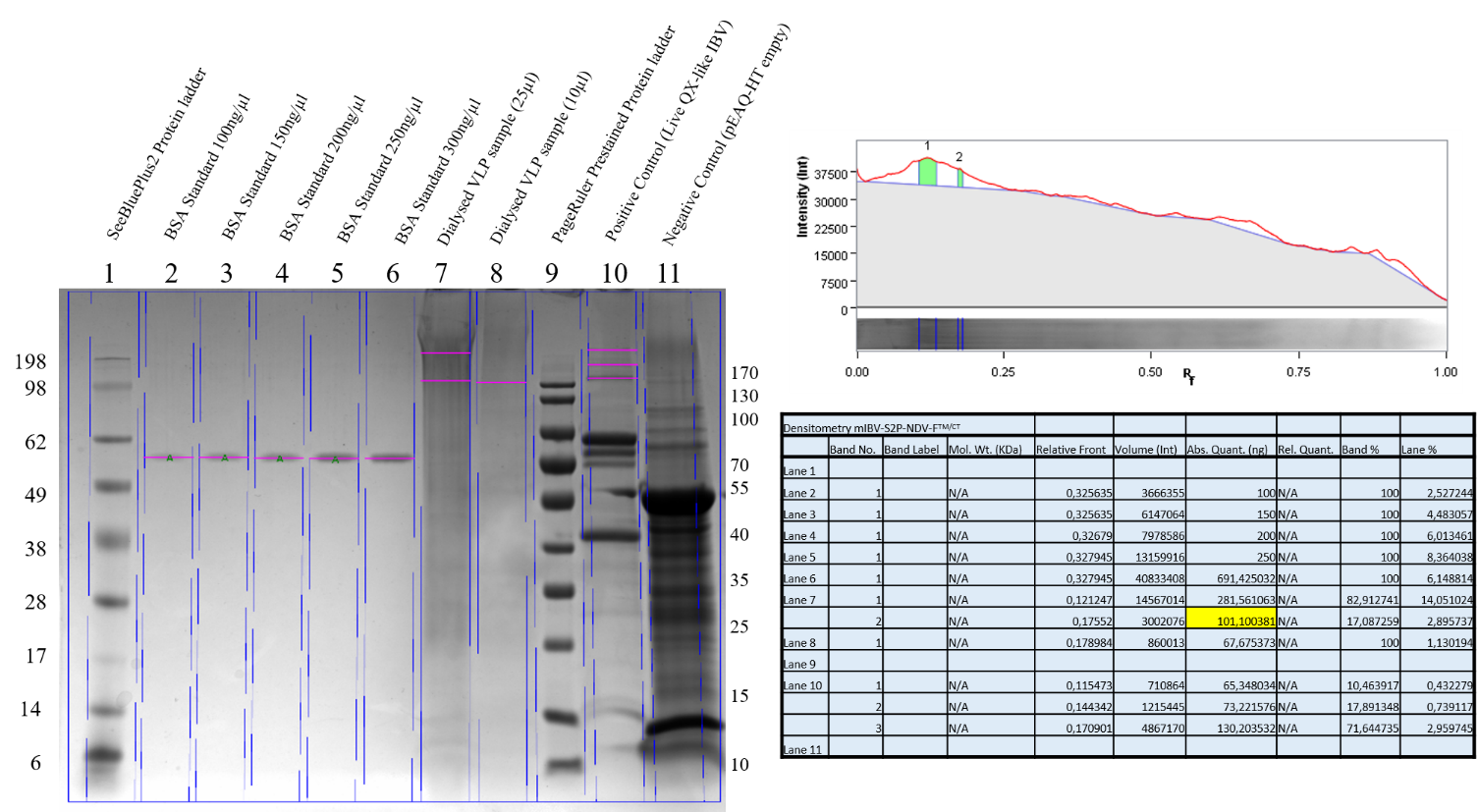

Supplement: S3 Fig — Lane 1: SeeBluePlus2 protein ladder; Lane 2: BSA Standard 100 ng/μl; Lane 3: BSA Standard 150 ng/μl; Lane 4: BSA Standard 200 ng/μl; Lane 5: BSA Standard 250 ng/μl; Lane 6: BSA Standard 300 ng/μl; Lane 7: Dialysed VLP sample (25 μl); Lane 8: Dialysed VLP sample (10 μl); Lane 9: PageRuler Prestained protein ladder; Lane 10: Positive control (Live QX-like IBV); Lane 11: Negative control (pEAQ-HT-empty). (DOCX) [file pone.0288970.s003.docx]

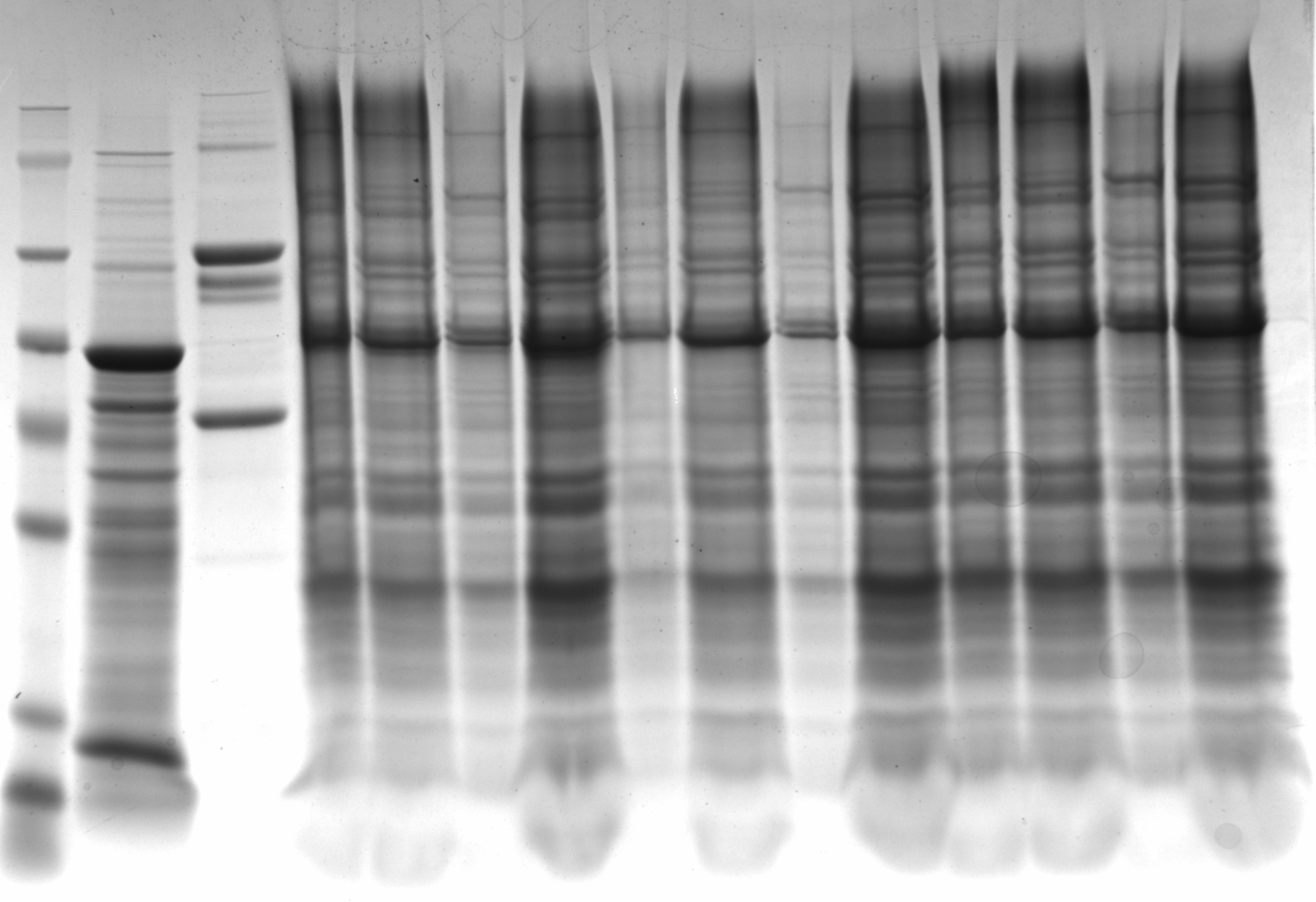

Supplement: S4 Fig — (PNG) [file pone.0288970.s004.png]

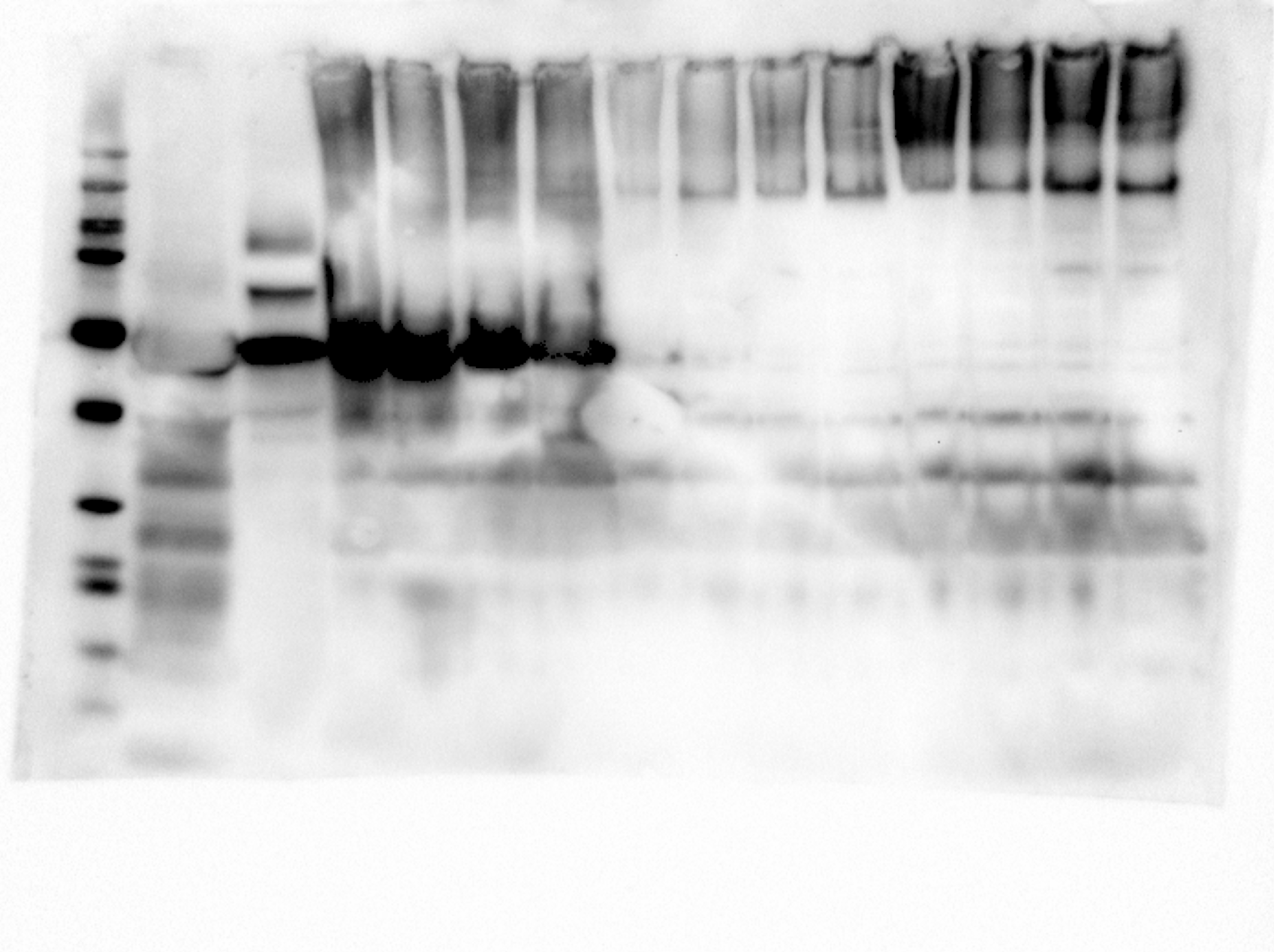

Supplement: S5 Fig — Lane 1: molecular weight marker; Lane 2: plant-expressed empty pEAQ-HT vector; Lane 3: purified live QX-like IBV strain ck/ZA/3665/11; Lanes 4–7: mIBV-S2P:M:E:N fractions 2 (lanes 4 and 6) and 3 (lanes 5 and 7) extracted in either PBS or bicine as indicated; Lanes 8–11: mIBV-S2P-IAV-H6TM/CT:M2 fractions 2 (lanes 8 and 10) and 3 (lanes 9 and 11) extracted in either PBS or bicine as indicated; Lanes 12–15: mIBV-S2P-NDV-FTM/CT:NDV Matrix fractions 2 (lanes 12 and 14) and 3 (lanes 13 and 15) extracted in either PBS or bicine as indicated. (TIF) [file pone.0288970.s005.tif]
