# Supplementary material for: Optimization of infectious bronchitis virus-like particle expression in Nicotiana benthamiana as potential poultry vaccines
Source: PLoS One. 2023 Jul 20;18(7):e0288970. doi: 10.1371/journal.pone.0288970 (PMC10358894; doi:10.1371/journal.pone.0288970)
Supplement: S1 Raw images — (PDF) [file pone.0288970.s006.pdf]

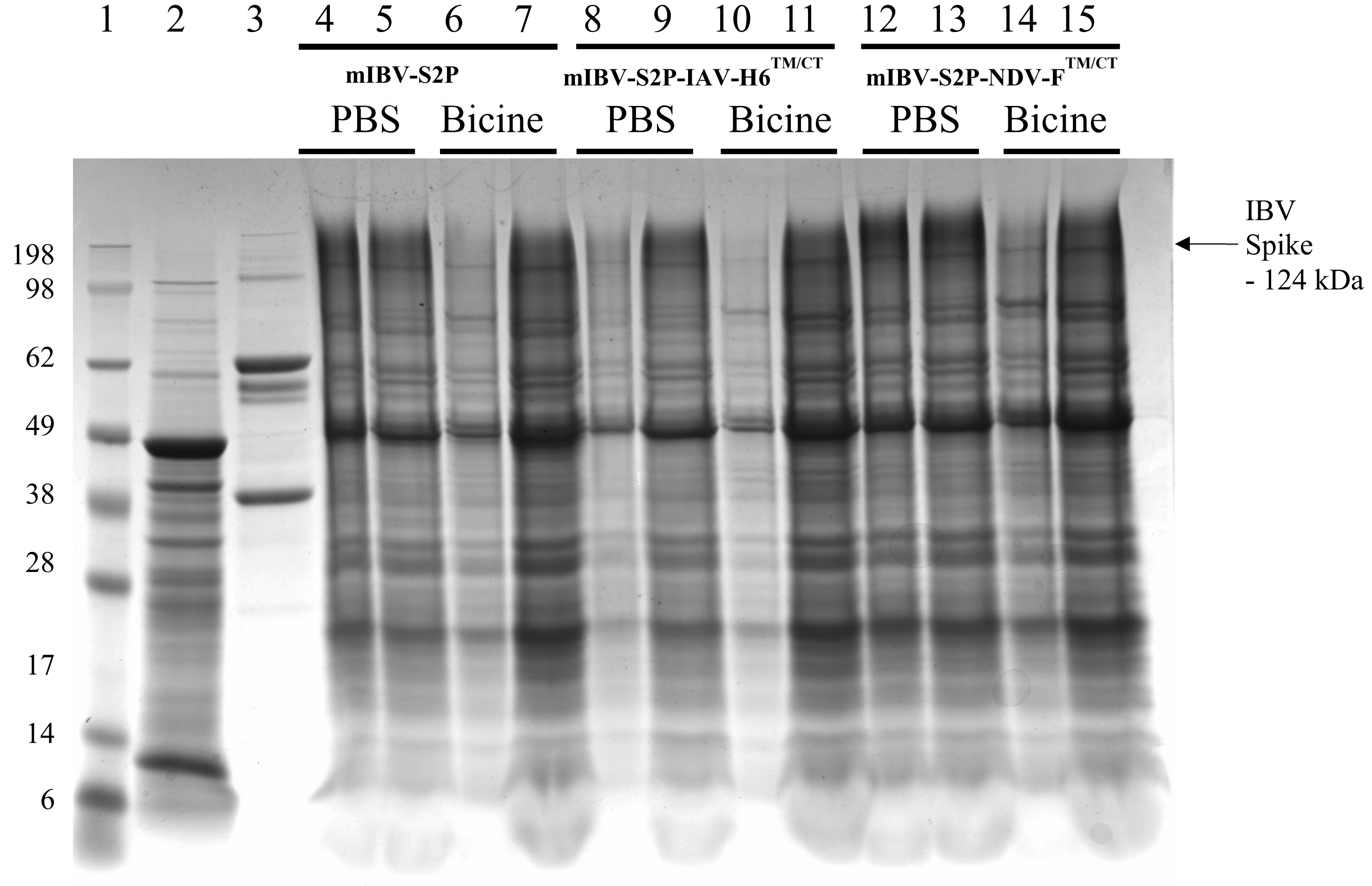

Original unaltered image for Fig1 (a), captured using ChemiDoc™ MP Imaging System (Bio-Rad). Mw marker: SeeBluePlus2 Prestained Protein Standard.

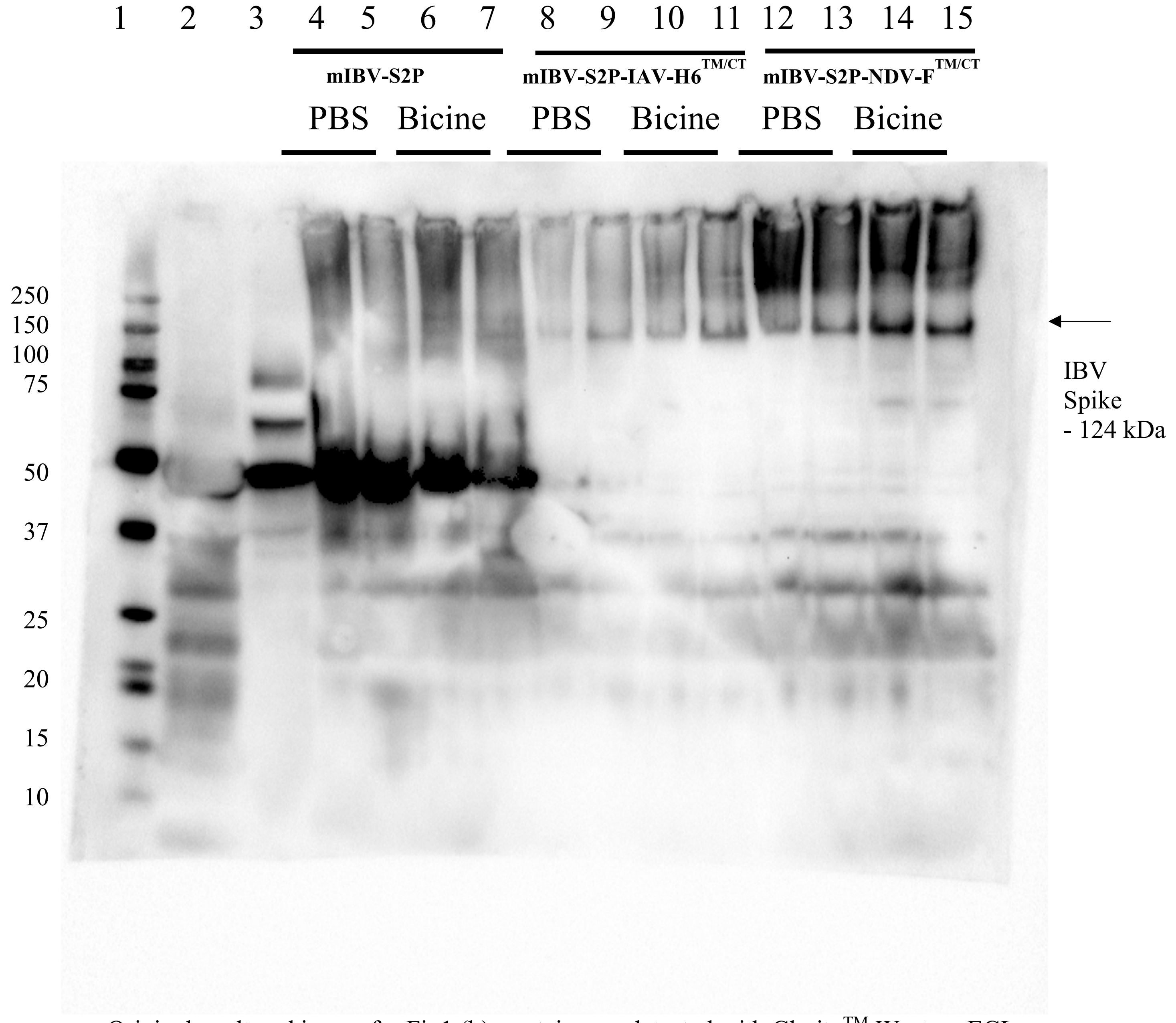

Original unaltered image for Fig1 (b), protein was detected with ClarityTM Western ECL chemilluminescence substrate (BioRad) and visualised using the ChemiDocTM MP Imaging System (Bio-Rad). Mw marker: Precision Plus Protein Western C Blotting Standard.
