# Supplementary material for: Optimization of infectious bronchitis virus-like particle expression in Nicotiana benthamiana as potential poultry vaccines
Source: PLoS One. 2023 Jul 20;18(7):e0288970. doi: 10.1371/journal.pone.0288970 (PMC10358894; doi:10.1371/journal.pone.0288970)
Supplement: Attachment — Submitted filename: Plos one manuscript review comment.docx [file pone.0288970.s007.docx]

In this manuscript, the authors describe a method by using Nicotiana benthamiana expression system to express IBV S protein and other viral proteins to produce virus like particles (VLPs). By co-expressing IBV S, N, M, E, or IBV S-NDV F (TMCM), NDV M, or IBV S-AIV H6(TMCM), AIV M2, the authors found the co-expressing group of IBV S-NDV F (TMCM), NDV M obtains the highest level of VLPs. This is a good attempt by using plant to produce IB VLPs as vaccine candidate, with lower cost and safer than the baculovirus insect cell expression system. The idea is novel; however, there is some issues need to be addressed in this study.

1. The results and figures legend interpret are not clear. Please check each figure’s interpret and give more detailed and clear information. For example, which antibody was applied for Fig. 1B? How was the HI result obtained in Fig. 3?
2. It seems the antibody for IBV is not sensitive for S protein detection. In Fig. 1B, there is no clear band for S protein in lane 3 (positive control). Also, it seems the membrane transfer has problem, the middle part (lane 8, 9, 10, 11, 12) membrane transfer is not successful, result in the white signal. Fig 1A, the arrow indicate S protein band also appears in the negative control (lane 2). Thus, it is not convincing to claim that the band is S protein. Usually, the glycol-S protein size is much bigger than 124 kDa. Please re-do the Western blot by using more specific S protein antibody. Current data is not convincing to show the successful expression of S protein. Moreover, please quantify the S protein containing VLPs.
3. The is no S antibody measurement in the chicken serum after immunization. Please use ELISA kit to measure the kinetic induction of S protein antibody in the serum.
4. Please do the QX strain attacking experiment to show the VLP protection efficiency, by using the H120 vaccination as control.
