# Supplementary material for: Optimization of infectious bronchitis virus-like particle expression in Nicotiana benthamiana as potential poultry vaccines
Source: PLoS One. 2023 Jul 20;18(7):e0288970. doi: 10.1371/journal.pone.0288970 (PMC10358894; doi:10.1371/journal.pone.0288970)
Supplement: Attachment — Submitted filename: Response to Reviewers.docx [file pone.0288970.s008.docx]

**Response to Reviewers**

**Journal Requirements:**

**When submitting your revision, we need you to address these additional requirements.**

**1. Please ensure that your manuscript meets PLOS ONE's style requirements, including those for file naming. The PLOS ONE style templates can be found at https://journals.plos.org/plosone/s/file?id=wjVg/PLOSOne_formatting_sample_main_body.pdf and 
https://journals.plos.org/plosone/s/file?id=ba62/PLOSOne_formatting_sample_title_authors_affiliations.pdf**

RESPONSE: The formatting has been revised as required.

**2. PLOS ONE now requires that authors provide the original uncropped and unadjusted images underlying all blot or gel results reported in a submission’s figures or Supporting Information files. This policy and the journal’s other requirements for blot/gel reporting and figure preparation are described in detail at https://journals.plos.org/plosone/s/figures#loc-blot-and-gel-reporting-requirements and https://journals.plos.org/plosone/s/figures#loc-preparing-figures-from-image-files. When you submit your revised manuscript, please ensure that your figures adhere fully to these guidelines and provide the original underlying images for all blot or gel data reported in your submission. See the following link for instructions on providing the original image data: https://journals.plos.org/plosone/s/figures#loc-original-images-for-blots-and-gels. 
  
In your cover letter, please note whether your blot/gel image data are in Supporting Information or posted at a public data repository, provide the repository URL if relevant, and provide specific details as to which raw blot/gel images, if any, are not available. Email us at plosone@plos.org if you have any questions**

RESPONSE: The original images have been uploaded as a supporting file (S1_raw_images).

**3. We note that you have included the phrase “data not shown” in your manuscript. Unfortunately, this does not meet our data sharing requirements. PLOS does not permit references to inaccessible data. We require that authors provide all relevant data within the paper, Supporting Information files, or in an acceptable, public repository. Please add a citation to support this phrase or upload the data that corresponds with these findings to a stable repository (such as Figshare or Dryad) and provide and URLs, DOIs, or accession numbers that may be used to access these data. Or, if the data are not a core part of the research being presented in your study, we ask that you remove the phrase that refers to these data**

RESPONSE: All the relevant data is actually presented, so the phrase has been replaced with an appropriate citation (lines 150-153), (lines 337-341), or was removed where we referred to our previous unpublished laboratory findings (lines 345-346).

**Reviewers' comments:**

**Reviewer's Responses to Questions**

**Comments to the Author**

**1. Is the manuscript technically sound, and do the data support the conclusions?**

**The manuscript must describe a technically sound piece of scientific research with data that supports the conclusions. Experiments must have been conducted rigorously, with appropriate controls, replication, and sample sizes. The conclusions must be drawn appropriately based on the data presented.**

**Reviewer #1: Partly**

**Reviewer #2: Partly**

**2. Has the statistical analysis been performed appropriately and rigorously?**

**Reviewer #1: I Don't Know**

**Reviewer #2: I Don't Know**

**3. Have the authors made all data underlying the findings in their manuscript fully available?

The** [**PLOS Data policy**](http://www.plosone.org/static/policies.action#sharing) **requires authors to make all data underlying the findings described in their manuscript fully available without restriction, with rare exception (please refer to the Data Availability Statement in the manuscript PDF file). The data should be provided as part of the manuscript or its supporting information, or deposited to a public repository. For example, in addition to summary statistics, the data points behind means, medians and variance measures should be available. If there are restrictions on publicly sharing data—e.g. participant privacy or use of data from a third party—those must be specified.**

**Reviewer #1: No**

**Reviewer #2: Yes**

**4. Is the manuscript presented in an intelligible fashion and written in standard English?**

**PLOS ONE does not copyedit accepted manuscripts, so the language in submitted articles must be clear, correct, and unambiguous. Any typographical or grammatical errors should be corrected at revision, so please note any specific errors here.**

**Reviewer #1: Yes**

**Reviewer #2: Yes**

**5. Review Comments to the Author**

**Please use the space provided to explain your answers to the questions above. You may also include additional comments for the author, including concerns about dual publication, research ethics, or publication ethics. (Please upload your review as an attachment if it exceeds 20,000 characters)**

**Reviewer #1: This paper describes the production of VLPs of IBV in plants using transient expression. Various different gene constructs are tested and the highest yielding one is used for an immunogenicity study in target animals. On the whole, this manuscript requires some modifications to improve clarity (especially with regards to the different constructs used and what Figure 1 shows). Finally the immunogenicity experiment described in figure 3 is lacking in any controls. More detailed points are raised below:**

**- The authors should add a figure that includes labelled diagrams for the constructs expressed in this paper.**

RESPONSE: The requested diagrams depicting constructs has been added as supplementary file (S3_Fig).

**- Figures S1 and S2 seems to have been swapped.**

RESPONSE: Thank you for picking this up, the figure titles have been amended accordingly.

**- Both supplementary figures appear to be TIFF image files, but they show sequence information. These files should therefore be in Word or PDF format so that readers can perform the “copy” function for BLAST searches, sequence analysis, etc.**

RESPONSE: Editable docx versions of these figures have been uploaded with the revised manuscript.

**- Figure 1: What is “fractions 2 and 3” referred to multiple times in the text and figure legend? Is this fractions from the sucrose cushion? If so what part of the gradient? The authors need to explain this clearly or show a diagram. Also the figure legend is unnecessarily confusing. If lanes 4-7 are from the same construct, then write something along the lines of “Lanes 4-7: mIBV-S2P:M:E:N fractions 2 (lanes 4 and 6) and 3 (lanes 5 and 7) extracted in either PBS or Bicine, as indicated”. Or add a line of labels in the figure itself above the wells for “fraction”, and another line for “construct”. In fact I would strongly recommend adding a “construct” line of labels to the images in Figure 1 regardless of changes to the figure legend. Same thing for lanes 8-11 and 12-15.**

RESPONSE: To clarify the concept of fractions, lines 176-178 were amended as follows: “0.5 ml fractions (successively labelled from fraction 1 upwards) were collected from the bottom of the 70 % sucrose gradient using a tube coupled to a peristaltic pump”. Figure legends were amended as suggested for clarity, and additional “construct” line was added above the wells in Fig1 as suggested.

**- Line 288-289: Gel densitometry is a rather poor method of protein quantification, a superior alternative would have been a modified Lowry assay or BCA assay. Because the authors chose to use gel densitometry, they need to show an image of the SDS-PAGE gel that was used for quantitation.**

RESPONSE: Yes, we agree that Lowry and BCA are superior methods for protein quantitation, but these can only be used to determine the total protein content. The protein extract here was intentionally partially purified because highly-purified VLP would drive up the production cost of such a veterinary vaccine, and it was important to test a preparation that would ultimately be commercially viable. The S-protein is the protective antigen, so we specifically needed an approximation of its concentration to calculate the vaccine dose, and we were therefore restricted to using densitometry of a PAGE gel. The densitometry gel has been added as a supplementary figure (S4_Fig).

**- Line 290: Is this expected? Does live IBV cause agglutination of CRBCs (I assume the answer is yes given how the subsequent immunogenicity assay was performed)? Do currently used IBV vaccines cause agglutination of CRBCs? The authors should indicate this for context and discuss.**

RESPONSE: Some live IBVs can agglutinate CRBCs in their native form but many (possibly the majority) cannot. Yes, this was expected because the native live IBV strain ck/ZA/3665/11 used in the design was unable to agglutinate CRBCs without neuraminidase pre-treatment, therefore we did not expect that the VLP would agglutinate CRBCs either but we tested it anyway. Live IBV strain ck/ZA/3665/11 could be treated with neuraminidase to enable it to be used as an antigen in the HI tests, and yes, we did try the neuraminidase treatment on the VLP without success, but the neuraminidase may not work on plant-produced proteins as with avian-produced antigens, and the heat inactivation step of the neuraminidase may also have a detrimental effect on the VLP.

Lines 296-302 were amended to better reflect the abovementioned, and with the reference of Ruano *et al*., 2000 included.

**- Line 299: All twenty samples tested negative for what?**

RESPONSE: Line 310 was amended to reflect that all twenty samples tested negative for the presence of IBV-specific antibodies.

**- Figure 3 and generally vaccination experiment: there are no controls in this experiment. This experiment should have a positive control (chickens vaccinated with a currently licensed vaccine) and a negative control (PBS + adjuvant, or, even better, chickens vaccinated with plant extract prepared and purified in the same way as the vaccine but without expression of the antigen + adjuvant). The authors should acknowledge this methodological weakness in the discussion and indicate that it makes any conclusion about immunogenicity preliminary rather than solid. I certainly hope that the live virus challenge that the authors mention will be performed with appropriate controls.**

RESPONSE: Thank you for the comment, but please bear in mind that there is no methodological weakness here because the clinical part of this study is not a vaccination experiment (which by definition entails a challenge with live virus), it is instead an immunogenicity experiment to verify that the VLPs do induce IBV-specific antibodies in the target species. Our Animal Ethics Committee was also insistent that this pilot be performed prior to the official vaccination study, not only to establish immunogenicity, but also toxicity. The pre-vaccination titres of the chickens served as the negative control here; there would be little point in including and ultimately sacrificing a group of non-immunized birds. We are compelled to reduce animal use. Yes, indeed we included appropriate control groups in the official vaccine study that followed this one. Lines 370-371 were modified to reiterate that this was an immunogenicity study that precedes the vaccine efficacy study.

**Reviewer #2: In this manuscript, the authors describe a method by using Nicotiana benthamiana expression system to express IBV S protein and other viral proteins to produce virus like particles (VLPs). By co-expressing IBV S, N, M, E, or IBV S-NDV F (TMCM), NDV M, or IBV S-AIV H6(TMCM), AIV M2, the authors found the co-expressing group of IBV S-NDV F (TMCM), NDV M obtains the highest level of VLPs. This is a good attempt by using plant to produce IB VLPs as vaccine candidate, with lower cost and safer than the baculovirus insect cell expression system. The idea is novel; however, there is some issues need to be addressed in this study.**

**1. The results and figures legend interpret are not clear. Please check each figure’s interpret and give more detailed and clear information. For example, which antibody was applied for Fig. 1B? How was the HI result obtained in Fig. 3?**

RESPONSE: Figure 1’s legend has been amended for clarity. Primary and secondary antibody now included in the figure legend, details of which can be found in the methodology, lines 182-192). Lines 242-247 describe the haemagglutination inhibition test (with reference to WOAH, 2019 for the standard method) used to obtain the HI results.

**2. It seems the antibody for IBV is not sensitive for S protein detection. In Fig. 1B, there is no clear band for S protein in lane 3 (positive control). Also, it seems the membrane transfer has problem, the middle part (lane 8, 9, 10, 11, 12) membrane transfer is not successful, result in the white signal. Fig 1A, the arrow indicate S protein band also appears in the negative control (lane 2). Thus, it is not convincing to claim that the band is S protein. Usually, the glycol-S protein size is much bigger than 124 kDa. Please re-do the Western blot by using more specific S protein antibody. Current data is not convincing to show the successful expression of S protein. Moreover, please quantify the S protein containing VLPs.**

RESPONSE: Yes, the S protein was not detected in the positive control. The most likely reason for this was that the quantity of S protein in the purified virus used for the positive control was low. The flock that the protein was purified from was also immunized with Mass-type IBV vaccines, and the level of cross-protection is low between the spike protein from different serotypes (Cavanagh, 2003). This is already mentioned in lines 265-268.

While a similar sized- band appears in the negative control, this is most likely a plant protein of a similar size. Most importantly, the corresponding bands in the relevant lanes, were excised from the gel (in lanes 5, 9, and 13, correlating to each construct) were analysed by LC-MS/MS (peptide sequencing), which confirmed the presence of the IBV spike protein in high enough quantities to confidently confirm that the S protein is present in those bands. The SDS-PAGE, Western blot, and LC-MS/MS results taken together allow us to confidently make the conclusion that the S-protein was well-expressed in the plants, and this was also subsequently verified by the inducement of IBV S-specific antibodies in the immunogenicity study in chickens. The gel used for densitometry quantification of the VLP S-protein concentration has been included as a supplementary figure (S4_Fig).

**3. The is no S antibody measurement in the chicken serum after immunization. Please use ELISA kit to measure the kinetic induction of S protein antibody in the serum.**

RESPONSE: There is no validated commercial ELISA available that measures IBV S-antibodies, presumably because of the vast number of IBV serotypes present globally and the propensity of IBV for antigenic drift. Developing and validating an S-specific ELISA for the QX-like virus on which the VLP designs were based was far beyond the scope of this study. Therefore, only HI could be used to measure specific S-antibody responses.

**4. Please do the QX strain attacking experiment to show the VLP protection efficiency, by using the H120 vaccination as control.**

RESPONSE: We are uncertain about what the reviewer means by an “attacking” experiment, but thought that he/she is referring to virus neutralization tests (with the H120 vaccine). Please bear in mind that the clinical part of this study was designed and intended only as an immunogenicity experiment, to demonstrate that the VLPs were immunogenic and non-toxic in the target species. We verified both, and in the vaccine study that follows this one, we officially evaluated the efficacy of the VLPs and the ability of the vaccine to reduce virus shedding and protect against disease; no further changes made.
