# Supplementary material for: Optimization of infectious bronchitis virus-like particle expression in Nicotiana benthamiana as potential poultry vaccines
Source: PLoS One. 2023 Jul 20;18(7):e0288970. doi: 10.1371/journal.pone.0288970 (PMC10358894; doi:10.1371/journal.pone.0288970)
Supplement: Attachment — Submitted filename: Response to reviewers and editor 260523.docx [file pone.0288970.s009.docx]

**Response to reviewers and editor**

Whilst we are enormously grateful to Dr. Ying for the time expended on this review, we unfortunately seem to have reached an impasse, and I explain below how and why this reviewer’s three remaining objections lack merit. I have copied Dr. Ying’s comments in boldface, with our responses below.

**1. The author should re-perform the Western blot in Fig 1b and get better quality figure.**

**The current Western blot figure quality is not acceptable, as there is no clear S protein**

**band, even in the positive control (lane 3).**

Ms. Sepotokele did repeat the Western blot several times, but she was unable to improve the quality. The S protein band, although faint, is still clearly visible. The figure we present is the best we can do at this time. It would be a shame to reject this novel work for this reason.

As we explained to the reviewer in our previous responses, included here for your benefit, the

reason for the faintness of the S-protein band in the Western blot is attributed to the fact that (a) the plant extract containing the VLPs is only partially purified and plant proteins cross-react nonspecifically with chicken antiserum (chickens are fed plant matter, so this is to be expected) and (b) that the only available IBV-positive antisera was collected from commercial chickens that were immunized with heterologous IBV vaccines, not the QX-like variant our VLP is based on. Thus, repeating the Western blot over and over again is not going to increase the intensity of the S-protein band whatsoever.

Finally, as we previously rebutted, peptide mass fingerprinting on this exact band confirmed that it is indeed the S-protein.

Alternatively, if the acceptance of the manuscript comes down to this minor issue, we could remove this Western Blot entirely, report it as “data not shown”; the peptide mass fingerprinting results are already included as supplemental data. Please let us know if this would be acceptable?

**2. S antibody measurement in the chicken serum after immunization is necessary.**

We already explained this point in the prior revision, the hemagglutinin inhibition (HI) test

specifically measures S antibodies, so we did indeed measure the S antibodies in the chickens after immunization; it is very strange that the reviewer won’t accept this fact.

**3. The QX strain infection experiment after immunization is necessary to show the VLP**

**protection efficiency, by using the H120 vaccination as control. As the title of this manuscript is “Optimization of Infectious Bronchitis virus-like particle expression in Nicotiana benthamiana as potential poultry vaccines”. The study should include experiments to show the VLP as potential vaccines. The successful expression of S protein and HI experiment are not enough to demonstrate it is potential vaccines.**

We completely disagree with Dr Ying’s view that successful expression of the S protein, and indeed the proof we presented in the study that S-specific antibodies are raised in chickens immunized with this VLP (as shown by HI), are insufficient to demonstrate the VLP’s potential as a vaccine.

It is very well known that the levels of coronavirus S-protein antibodies provide a predictive

correlate of protection in the host. With regard to the first sentence, the H120 vaccine is one of

many applied in the poultry industry globally, and is not a benchmark for establishing IBV vaccine efficacy. Furthermore, the use of the word “Potential” in the title is deliberate.

We also, in our prior rebuttal, carefully explained that this study, which focused on optimising the Gammacoronavirus VLP expression in planta (which, we point out again, is a **world first**) was followed up by a vaccine-challenge study. For your confidential information, we attach a copy of our submitted manuscript, to prove to you that these two manuscripts are intended to be complimentary studies. There was too much information to describe all our processes and results in a single paper, hence our decision to split it into an optimisation study (for a “potential” vaccine), followed by the in vitro vaccine-challenge study with the optimised VLP.

To support our manuscript’s acceptance to PLOS ONE in its current form, we must point out that two expert reviewers for PLOS ONE have already approved this manuscript. Ms Sepotokele is a PhD candidate, and her thesis in which this work was presented has already been examined and passed. I can confidentially disclose that the data in this manuscript was additionally reviewed and approved by two external examiners (and an internal examiner). The two external examiners were Prof. Janet Daly from Nottingham University, who is a leading world expert in veterinary vaccine development (including plant VLPs), and Prof Inge Hitzeroth from the University of Cape Town, who is a leading expert in plant-based vaccine development.

In conclusion, I am respectfully requesting that all of the abovementioned reasons and explanations are taken into account, and that you exercise your discretion as the academic editor to make a final decision on whether or not our manuscript will be accepted to PLOS ONE in its current form.

Yours sincerely,

Prof. Celia Abolnik

NRF-DSI SARChI- Poultry Health and Production

PhD Promotor

Email: celia.abolnik@up.ac.za
