# Supplementary material for: Optimization of infectious bronchitis virus-like particle expression in Nicotiana benthamiana as potential poultry vaccines
Source: PLoS One. 2023 Jul 20;18(7):e0288970. doi: 10.1371/journal.pone.0288970 (PMC10358894; doi:10.1371/journal.pone.0288970)
Supplement: Attachment — Submitted filename: Response to Reviewers PLOS One 260623.docx [file pone.0288970.s010.docx]

**Response to Reviewers PLOS One**

**Reviewer #1: I was happy with the authors' modifications after the first round of reviews. I'm still ok with this manuscript being published.**

RESPONSE: Thank you.

**Reviewer #3: This is a resubmission of a manuscript that has already been reviewed twice. From the correspondence associated with this latest version, it appears that although Reviewer 1 for second revision was happy with the changes made, Reviewer 2 (Dr. Liao Ying) was still not satisfied. Given that this manuscript has already been extensively reviewed by others, I will concentrate on the outstanding issues raised by Dr. Ying.**

**1. I disagree with the comment of Dr. Ying that there are no clear bands in the western blot in Fig. 1B. The bands corresponding to the full-length S protein are clearly visible in lanes 8-15, as are higher molecular weight forms which are commonly seen with multimeric proteins. The fact that the positive control does not give a clear band representing full-length S protein is really neither here nor there - it would only be an issue if no bands were seen in the test samples.**

**That said, I feel the authors do not do themselves any favours by not mentioning the absolutely obvious band at around 50kDa in lanes 4-7. This is also present in the positive control. This most probably represents the cleaved form of the S protein (S1 and/or S2) and such cleavage has been reported previously with plant expressed SARS-CoV-2 S protein (see Fig 2 oof reference [30]. The fact that this cleavage appears to be greatly reduced with the modified S protein (lanes 8-15) validates the authors' modification strategy and this should highlighted. At any rate the appearance of the 50kDa product must be discussed.**

RESPONSE: The bands present in lanes 4-7 at around 50 kDa (Western blot Fig. 1B), were thought to correlate to the highly conserved 45 kDa IBV Nucleocapsid protein, which would be present in the positive control because the positive control contains it. The reason this protein was only observed in lanes 4-7, was because those lanes contained the mIBV-S2P VLPs that had been produced by co-infiltrating with the IBV Membrane, Envelope, and Nucleocapsid proteins. It is not present in lanes 8-15 because constructs mIBV-S2P-IAV-H6^TM/CT^ (8-11) and mIBV-S2P-NDV-F^TM/CT^ (12-15) were not co-infiltrated with the IBV M, E, and N proteins, but co-infiltrated with accessory proteins corresponding to IAV and NDV instead.

This was discussed in lines 257-260. No further changes made.

**Two more minor points:**

**I do think that the S protein can be described as being comprised of two "subunits". This implies separately synthesised polypeptides which - the usual description is two "domains".**

RESPONSE: “subunit/s” has been replaced with “domain/s” where necessary.

**Fig.1 would greatly benefit from having a simple version of the constructs shown as an addition panel (C?). This would greatly assist the reader and avoid the necessity for constantly cross-referencing with the SI.**

RESPONSE: Thank you for the suggestion. Fig 1. has now been amended to include an additional panel C, which contains the constructs. Fig S3 has accordingly been removed to avoid repetition.

**2. I have no problem with the immunological analysis as presented in the revised ms.**

RESPONSE: Thank you.

**3. From the correspondence, it is clear the authors have additional information about the immunological properties of their VLPs which they wish to present in a separate MS. Though I appreciate their desire to present the work in two papers, I really thing the data would be better combined, especially as the current MS is so short (only 3 figures!). I think one MS would make far greater impact overall and I urge the authors to consider this.**

RESPONSE: While we do understand this concern, the present manuscript covers the extensive work and technical details (multiple constructs and combinations of accessory proteins) that went into obtaining successful IBV VLP expression for the first time (which is novel), and we demonstrated the immunogenicity in chickens to prove that the S protein is properly folded and presented on the VLP. The second manuscript is a classical vaccine-efficacy study that is more suited to a veterinary journal, and we felt that with a single paper those reviewers might consider the in-depth description of how the VLP was developed of less interest to veterinarians and poultry scientists, and consequently request that the developmental data (that is of interest to plant scientists designing such VLPs) be removed. For this reason, we deemed it necessary to publish these aspects of the studies separately.

The second manuscript that was submitted to “Poultry Science” has already been reviewed and we are busy preparing a revised version that incorporates those reviewers’ suggested changes, therefore we are unwilling to withdraw it at this stage in order to incorporate the data here (along with the other reason we describe above). We hope that Prof. Lomonossoff is satisfied with this explanation.
